# Utilizing Shear Wave Elastography for the Evaluation of Ocular Involvement in Systemic Sclerosis

**DOI:** 10.3390/diagnostics15101227

**Published:** 2025-05-13

**Authors:** Mehmet Kök, Ayşe Ayan, Mehmet Emin Arayici, Sinan Ülgen

**Affiliations:** 1 Department of Internal Medicine, Antalya Training and Research Hospital, University of Health Sciences, Antalya 07100, Turkey; 2 Department of Rheumatology, Antalya Training and Research Hospital, University of Health Sciences, Antalya 07100, Turkey; drayseayan@yahoo.com.tr; 3 Department of Biostatistics and Medical Informatics, Faculty of Medicine, Dokuz Eylül University, Inciralti-Balcova, Izmir 35340, Turkey; mehmet.e.arayici@gmail.com; 4 Department of Radiology, Antalya Training and Research Hospital, University of Health Sciences, Antalya 07100, Turkey; sinanulgen@gmail.com

**Keywords:** systemic sclerosis, ocular elasticity, ultrasound elastography, shear wave

## Abstract

**Background:** Several imaging studies have confirmed ocular involvement in systemic sclerosis (SSc). However, elastography has not yet been used for this purpose in the literature. Thus, this study aimed to evaluate ocular involvement in SSc using shear wave elastography (SWE). **Methods:** This study included 29 SSc patients and 30 age- and sex-matched healthy controls. All participants underwent independent ophthalmological evaluations by two ophthalmologists. Subsequently, SWE was used to evaluate the retina–choroid–sclera (RCS), optic disc (OD), optic nerve (ON), and retrobulbar adipose tissue (RBFT) of the right eye. The median shear wave elasticity (kPa) and velocity (m/s) values were automatically calculated using the ultrasound device’s integrated software. **Results:** The elasticity and velocity values of RBFT in SSc patients were significantly higher than those in the control group. However, no notable differences were observed in other analyzed areas. A strong association was found between digital ulcers and velocity values of the RCS, while no significant differences were noted for other parameters. **Conclusions:** This study revealed increased stiffness in the RBFT of SSc patients. To our knowledge, this is the first evidence suggesting that SSc can affect RBFT. Further studies are required to confirm this finding and investigate its link to the disease. Additionally, we found a strong association between digital ulcers and increased RCS stiffness. Using SWE for the first time, we have demonstrated that microcirculatory disruption in SSc extends beyond the skin and can affect multiple tissues simultaneously.

## 1. Introduction

Systemic sclerosis (SSc) is a chronic, multisystem disorder characterized by immune system activation, extensive vasculopathy, and progressive fibrosis, and presenting with a wide range of clinical symptoms [1,2]. Clinically, SSc is classified into two subtypes: limited cutaneous systemic sclerosis (lcSSc), which is characterized by predominant skin involvement with relatively less internal organ fibrosis, and diffuse cutaneous systemic sclerosis (dcSSc), in which internal organ involvement often follows rapidly after the onset of skin lesions [3]. A hallmark feature of SSc is microvascular dysfunction, affecting not only the skin but also internal organs such as the heart, lungs, kidneys, gastrointestinal tract, and eyes [4]. Raynaud’s phenomenon (RP) is often the earliest and most consistent manifestation. Microvascular injury, characterized by endothelial disruption, inflammatory cell infiltration, and vessel obliteration, can eventually lead to tissue ischemia, organ dysfunction, and capillary loss [5].

Ocular involvement is common in SSc, affecting both the anterior and posterior segments. Documented ocular manifestations include optic neuropathy, primary retinopathy, choroidopathy, keratoconjunctivitis sicca, anterior uveitis, and normal tension glaucoma [6,7]. The susceptibility of the eye to pathological changes is largely attributed to the high collagen content of ocular tissues and the retinal microcirculatory system. While fibrotic processes primarily involve the anterior segment, microvascular changes predominantly affect the retina and choroid [8]. Though visual impairment is rare and posterior segment involvement is often asymptomatic, mild retinal vascular damage has been observed in 34–55% of SSc patients, sometimes preceding systemic or cutaneous symptoms [9,10]. For this reason, the early detection of ocular microcirculatory changes may assist in earlier diagnosis and management.

Ultrasound examination is one of the most widely employed diagnostic imaging methods in contemporary medical practice, maintaining a central role within diagnostic frameworks. Its non-invasive nature, relatively low cost, and widespread accessibility have made it the most commonly utilized diagnostic imaging method across various medical disciplines. Elastography is one of the latest advancements in ultrasound technology and was first introduced in the early 1980s [11]. Ultrasound elastography identifies alterations in tissue elasticity resulting from specific pathological or physiological processes [12]. The assessment of tissue elasticity and stiffness has proven valuable in the differential diagnosis of tumors, inflammation, and normal tissues. This technique has been applied to the evaluation of the breast, thyroid, prostate, cervix, liver, heart, musculoskeletal system, and selected lymph node groups [12,13]. In 2010, the first study to investigate the use of elastography for assessing ocular structures demonstrated its feasibility [14], and subsequent studies have applied this technology to orbital diseases such as glaucoma, optic neuritis, and multiple sclerosis [15,16,17].

Strain wave elastography, acoustic radiation force impulse imaging, shear wave elastography (SWE), and transient elastography represent distinct elastography methods. SWE offers several advantages, including providing true quantitative elasticity measurements (in kilopascals) and eliminating the need for external compression, making it, in particular, more suitable for internal organ assessment [12,18].

This prospective cross-sectional study aimed to examine the impact of SSc on the retina–choroid–sclera (RCS), optic nerve (ON), optic disc (OD) and retrobulbar fat tissue (RBFT) using the SWE technique in patients with SSc. Additionally, we aimed to explore potential correlations between SWE values and clinical parameters such as disease duration, subtype, and severity.

## 2. Materials and Methods

### 2.1. Subject

This prospective cross-sectional study was conducted at Antalya Education and Research Hospital from January 2021 to January 2022. The study involved 29 patients diagnosed with SSc according to the American College of Rheumatology criteria, alongside 30 age- and sex-matched healthy controls without any ocular or systemic diseases [19]. In the literature, both the onset of RP and the first manifestation of non-Raynaud’s symptoms are recognized as reference points for determining disease duration in systemic sclerosis. Accordingly, in our study, we evaluated disease duration by considering both the onset of RP and the onset of the first non-Raynaud’s symptom as distinct starting points.

In our rheumatology clinic, the follow-up protocol for patients with SSc includes routine assessments for organ involvement, particularly of the lungs, liver, and kidneys, at designated intervals. We reviewed the follow-up records of all included patients. Additionally, we examined our patient record system to assess the presence of specific autoantibodies, including anti-Scl-70 and anti-centromere antibodies. Some individuals were found to have interstitial lung disease, pulmonary hypertension (PHT), and gastroesophageal reflux. However, no cases of cardiac or renal complications were identified.

The following patients were excluded: (1) those with connective tissue diseases other than SSc; (2) those with hypertension; (3) those with comorbid conditions affecting vasculopathy, such as diabetes mellitus, systemic hypertension (defined as systolic blood pressure above 140 mm Hg and/or diastolic blood pressure above 90 mm Hg or requiring antihypertensive medication), cardiovascular disease, etc.; (4) smokers.

In our study, digital ulcers (DUs) were clinically identified (past or present) by the attending physician as lesions located on the digits characterized by a visible depth and a disruption in the integrity of the epithelial surface and abnormal nailfold capillaroscopy findings in the fingers. Nailfold capillary changes alone were not considered sufficient to diagnose DUs [20]. All of our patients diagnosed with DUs demonstrated abnormal nailfold capillaroscopy findings, along with a history and clinical presentation consistent with DUs. None of the patients in our study had active DUs at the time of assessment. The diagnosis of PHT was established in accordance with the latest clinical guidelines [21].

Two ophthalmologists performed a comprehensive ocular examination. Additional ocular exclusion criteria are outlined in the “Ophthalmological Examination” subsection of the Section 2.

The present study received ethical approval from the Ethics Committee of the University of Health Sciences, Antalya Training and Research Hospital (Approval Date: 4 June 2020; Reference No: 8/3). Written informed consent was obtained from all participants before their inclusion in the study. The research was conducted in full accordance with the principles outlined in the Declaration of Helsinki.

### 2.2. Ophthalmological Examination

A total of 59 individuals were enrolled in this study. Each subject underwent a thorough ophthalmological evaluation, which included best-corrected visual acuity (BCVA) assessment using the decimal scale, intraocular pressure (IOP) measurement, axial length (AL) determination, slit-lamp biomicroscopy, and dilated fundus examination. Color fundus photography and fundus autofluorescence imaging were performed using the Visucam NM/FA system (Carl Zeiss Meditec AG, Jena, Germany). Fluorescein fundus angiography (FFA) was performed in cases where ocular involvement was suspected or clinically indicated. Two retina specialists, blinded to the participants’ clinical status, independently evaluated all imaging data. SWE evaluations were completed on the same day as the ophthalmic examinations. During the fundus examination, two ophthalmologists assessed SSc patients for increased vascular tortuosity, focal or generalized arteriolar narrowing, arteriovenous notching, severe exudation, microhemorrhages, and pigment epithelial changes in the retina. Patients presenting with at least two of these findings were classified as having retinopathy [22]. During the fundus examination, individuals presenting with retinopathies unrelated to SSc, including, but not limited to, age-related macular degeneration, diabetic retinopathy, vascular sheathing or attenuation, chorioretinal scarring, retinal pigment epithelium mottling, glaucomatous optic nerve alterations, optic nerve edema, or pallor, were excluded from the study. Furthermore, participants with a history of intraocular surgery, laser therapy, uveitis, high myopia, or suboptimal image quality were not included. Patients diagnosed with glaucoma or those with a known family history of glaucoma were also excluded.

### 2.3. Orbital SWE Examination

All participants in the study underwent evaluation using a Toshiba Aplio 500 ultrasound system (Tokyo, Japan) equipped with a 6–18 MHz linear transducer. Examinations were performed exclusively on the right eye of each subject. This decision was made to ensure standardization in the procedures and to prevent possible natural variations between eyes from complicating the analysis. At the same time, we aimed to increase patient comfort by reducing the procedure time.

Previous studies have shown that when no significant anatomical or elasticity differences are expected between the eyes, evaluation is usually performed on one eye (most commonly the right eye) [23,24,25]. Research indicates that ocular manifestations in systemic sclerosis commonly present with bilateral symmetry [26,27,28]. Therefore, assuming that the two eyes of the same participant would be similar, we assessed only the right eye of each participant.

During the procedure, patients were positioned supine with their eyes closed, and an appropriate amount of ultrasound gel was applied to the probe surface. The probe was carefully placed on the closed upper eyelid without applying excessive pressure. Throughout the examination, patients were instructed to keep their eyes closed and minimize ocular movements. All measurements were performed by a single radiologist with five years of experience in ultrasonographic imaging. Each measurement was taken three times, and the mean of the three values was recorded for analysis. In addition, analysis of variance of repeated elastography values in the same anatomical region was performed. The region of interest (ROI) cursor was sequentially positioned on the optic nerve head, retro-orbital nerve, sclera–retina complex, and retro-orbital adipose tissue, with tissue stiffness values automatically calculated and recorded.

**Figure 1:**
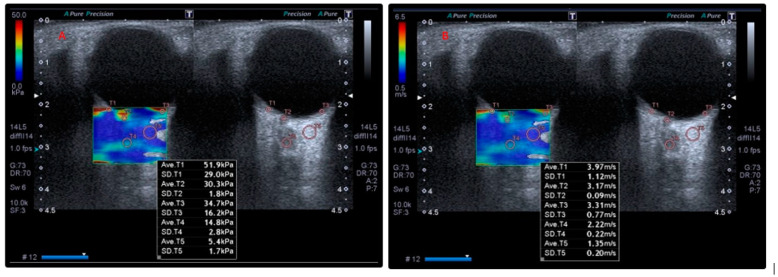
Elastography findings of a patient. Elasticity (kPa) (**A**) and velocity (m/s) (**B**) values obtained using shear wave elastography technique on retrobulbar adipose tissue (T5), optic disc (T2), optic nerve (T4), and retina–choroid–sclera (T1 and T3) in a patient with a diagnosis of scleroderma.

**Figure 2:**
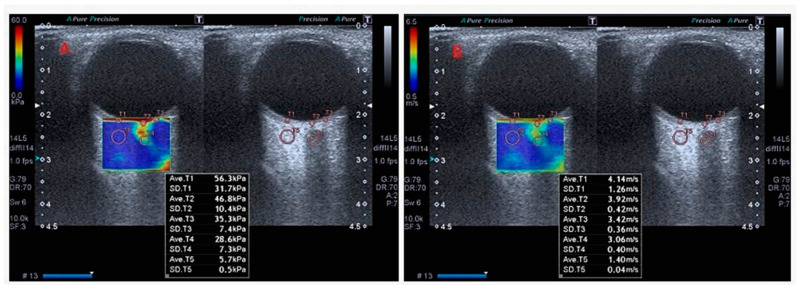
Elastography findings of a healthy control participant. Elasticity (kPa) (**A**) and velocity (m/s) (**B**) values obtained using shear wave elastography technique on retrobulbar adipose tissue (T5), optic disc (T2), optic nerve (T4), and retina–choroid–sclera (T1 and T3) in a patient with a diagnosis of scleroderma.

Measurements were taken by positioning the probe at an oblique angle in an area without vascular structures. SWE assessments were conducted within a region of interest (ROI) measuring 0.5 cm × 1.0 cm, using at least 10 focal points in the RBFT. The median values of shear wave elasticity (kPa) and velocity (m/s) were automatically calculated using the ultrasound device’s built-in software (Figure 1 and Figure 2).

To assess RCS measurements, two regions of interest (ROIs) were placed on both sides of the RCS adjacent to the optic disc, and the average of these two measurements was calculated. Circular areas of similar size were selected to ensure standardization across all patients, though slight adjustments were made to accommodate individual variations in tissue dimensions.

### 2.4. Statistical Analysis

In this study, the differences in elastography values between the SSc patient group and the healthy control group were evaluated. The elasticity and velocity values, including RCS, OD, ON, and RBFT, were measured for all participants. Additionally, the effects of disease type, the presence of IAH and DUs on these parameters were examined. Before conducting comparisons between the two groups, the distributions of the relevant variables within subgroups were analyzed to assess whether the normality assumption was met using multiple methods.

Three approaches were employed to determine whether the distributions followed a normal pattern. First, the Kolmogorov–Smirnov and Shapiro–Wilk tests were conducted, and if the probability value was greater than 0.05, the distribution was considered normal. Second, the skewness and kurtosis coefficients of the distributions were examined, and if these values were within the range of −2 to 2, the normality assumption was accepted. Finally, the Z-values of skewness and kurtosis coefficients were analyzed, with values between −1.96 and 1.96 being indicative of a normal distribution. Distributions that met at least two of these criteria were deemed to be normal, and, accordingly, the normality assumption was satisfied for all subgroups.

After confirming the normality assumption, independent sample’s t-tests were performed to compare elastography and velocity measurements between the two groups. Fisher’s exact test was used in the analysis of categorical data. Reproducibility of the SWE measurements was quantified by calculating intraclass correlation coefficients (ICC) with a two-way mixed-effects, absolute agreement model (ICC). For each intraclass correlation coefficient, a 95% confidence interval was calculated via Fisher’s z-transformation, thereby quantifying the precision of the reliability estimates. Receiver operating characteristic (ROC) curves were constructed to evaluate the diagnostic accuracy of SWE-derived elasticity values in discriminating patients from controls. The area under the curve (AUC) with corresponding 95% confidence intervals was estimated using the non-parametric DeLong method, and the associated *p*-value for the AUC was subsequently reported to quantify overall test performance. Furthermore, Pearson’s product-moment correlation coefficient was used to examine the relationships between elasticity and velocity values and FVC, DLCO, reflux, PHT, and disease duration. All computations were performed in IBM SPSS Statistics (version 30.0, IBM Corp., Armonk, NY, USA) and STATA software (v.18, College Station, TX, USA). Statistical significance was assessed using two-tailed *p*-values, with α set at 0.05.

## 3. Results

An analysis of the participants’ demographic characteristics revealed no statistically significant differences between the SSc group and the control group with respect to mean age, gender distribution, or BMI, with *p*-values of 0.104, 0.413, and 0.717, respectively. Other demographic and clinical characteristics of the patients are presented in Table 1.

A statistical summary of the repeated measurements for all elasticity and velocity parameters is provided in Table 2. The analysis across eight different parameters demonstrated that the mean coefficients of variation (CV) ranged from 0.49% to 1.50%. Specifically, the mean CV for elasticity measurements varied between 1.02% and 1.50%, whereas velocity measurements exhibited lower mean CVs, ranging from 0.68% to 0.84%. The lowest variability was recorded for elasticity values of the OD (0.49%), while the highest was observed for elasticity values of the RBF (1.50%). These consistently low CV values across all parameters suggest that the measurement techniques employed in this study have high reproducibility and reliability.

The SWE values for RCS, OD, ON, and RBFT in both the patient and control groups were analyzed and compared in terms of elasticity and velocity values. When comparing elasticity values, the mean elasticity value of the RBFT value in the SSc group (8.78 ± 2.75) was significantly higher than in the control group (6.08 ± 1.48), t(57) = 4.74, *p* < 0.001, d = 1.23). According to Cohen’s classification, this corresponds to a large effect size. No significant statistical association was observed between the elasticity values of the other analyzed areas (RCS, OD, ON) and the groups.

Similarly, when comparing velocity values, the mean velocity values of the RBFT value (1.60 ± 0.37) in the SSc group were significantly higher than in the control group (1.44 ± 0.21), t(57) = 2.13, *p* = 0.037, d = 0.53). This effect size indicates a moderate effect. No significant statistical association was observed between the velocity values of the other analyzed areas (RCS, OD, and ON) and the groups (Table 3).

We analyzed SWE values based on the type of SSc involvement (diffuse vs. limited). Regarding elastography measurements, no significant differences were observed between the two groups in the elasticity values of the RCS [t(27) = 0.20, *p* = 0.841], elasticity values of the OD [t(27) = 0.19, *p* = 0.852], elasticity values of the ON [t(27) = 0.31, *p* = 0.762], and elasticity values of the RBFT [t(27) = 0.76, *p* = 0.456].

Similarly, the velocity measurements showed no significant differences between patients with diffuse and limited SSc for the velocity values of the RCS [t(27) = 0.37, *p* = 0.718], velocity values of the OD “[t(27) = 0.97, *p* = 0.340], velocity values of the ON [t(27) = 0.45, *p* = 0.657], and velocity values of the RBFT [t(27) = 0.04, *p* = 0.972]. These findings indicate that elastography and velocity measurements in SSc patients do not significantly vary based on disease type (Table 4).

Patients were categorized into two groups based on the presence or absence of interstitial lung disease (ILD), and their SWE values were compared. The analysis revealed no statistically significant differences between the two groups. The relationship between patients’ FVC and DLCO values and their SWE measurements was analyzed. Among the elastography variables, the elasticity values of the RCS exhibited a weak negative correlation with FVC (r = −0.30) and DLCO (r = −0.22). Similarly, the elasticity values of the OD demonstrated a slight negative correlation with FVC (r = −0.22) and DLCO (r = −0.18). The elasticity values of the ON and RBFT showed negligible, non-significant correlations with both FVC and DLCO. When assessing velocity variables, the velocity of the RCS exhibited a moderate negative correlation with FVC (r = −0.33) and DLCO (r = −0.28), though these associations were not statistically significant. Additionally, the velocity values of the OD, ON, and RBFT demonstrated very weak, non-significant correlations with FVC and DLCO. These results indicate that there is no significant relationship between elastography or velocity measurements and FVC or DLCO in patients with SSc (Table 5).

Additionally, the relationships between disease durations (post-first RP disease duration and post-non-Raynaud’s symptoms disease duration) and elastography and velocity variables were analyzed. The results indicated that none of the variable pairs showed statistically significant correlations. The highest correlation with post-first RP disease duration was observed for elasticity of the RCS (r = 0.22) and velocity of RBF (r = −0.28). Similarly, the most notable correlation with post-non-Raynaud’s symptoms disease duration was found for the velocity of RBF (r = −0.32). Overall, elastography and velocity parameters demonstrated weak and non-significant associations with disease durations (Table 5).

An analysis was conducted to determine whether elastography measurements varied based on the presence of pulmonary PHT. The findings revealed no statistically significant differences between the groups across all measured variables (*p* > 0.05).

Elastography values were also evaluated according to the presence of oesophageal reflux. A significant difference was observed between the groups only in the elasticity values of the OD variable, t(27) = 2.71, *p* = 0.012, d = 1.01. According to Cohen’s effect size classification, this effect size indicates a large difference (Table 6).

Patients were categorized into two groups based on the presence of DUs, and their SWE values were compared. Regarding elastography measurements, patients with DUs had significantly higher elasticity RCS values (M = 104.41, SD = 21.01) compared with those without DUs (M = 75.88, SD = 18.56) [t(27) = 3.83, *p* = 0.001, d = 0.87], indicating a large effect size according to Cohen’s classification. Other elastography parameters, including elasticity OD [t(27) = 1.87, *p* = 0.072], elasticity ON [t(27) = 1.71, *p* = 0.099], and elasticity RBF [t(27) = 0.43, *p* = 0.674] did not show statistically significant differences between the groups. In terms of velocity measurements, velocity RCS was also significantly higher in the DUs group (M = 6.11, SD = 0.77) compared to the non-DUs group (M = 4.99, SD = 0.78) [t(27) = 3.87, *p* = 0.001, d = 0.88], again indicating a large effect size. However, velocity OD [t(27) = 1.53, *p* = 0.137], velocity ON [t(27) = 1.70, *p* = 0.101], and velocity RBF [t(27) = 0.55, *p* = 0.560] did not differ significantly between the groups. These findings suggest that the elasticity RCS and velocity RCS values are significantly elevated in patients with digital ulcers, while other elastography and velocity parameters do not significantly differ based on DU status (Table 7).

Finally, the performance of elastography values in the diagnosis of SSc was evaluated (Table 8). The best performance was found in the elasticity values of the RBF. The AUC value for the elasticity of RBF variable demonstrated a high discriminative power with 0.870 (0.782–0.952), while 79.0% sensitivity and 77.0% specificity rates were achieved at a cut-off value of 7.35 (*p* < 0.001).

## 4. Discussion

In this study, SWE was employed to evaluate RCS, ON, OD, and RBFT in patients diagnosed with SSc. The elasticity and velocity measurements of the RBFT were significantly higher in the SSc group compared with the control group, indicating increased tissue stiffness. However, no statistically significant differences were identified between the groups for the other examined regions. Furthermore, the elasticity values of the RBFT demonstrated significant diagnostic performance in the detection of SSc with a sensitivity of 79% and specificity of 77%. A strong correlation was found between OD elasticity and gastroesophageal reflux as well as between the presence of DUs and increased RCS stiffness.

A range of visual disturbances has been reported in SSc, affecting both the anterior and posterior segments of the eye. Documented manifestations include optic neuropathy, primary retinopathy, choroidopathy, keratoconjunctivitis sicca, anterior uveitis, and normal tension glaucoma [6,7,22]. Since the choroid is the most vascularized part of the eye, SSc-related abnormalities are expected to have a significant impact. However, the literature presents conflicting findings. Early studies found no link between SSc and retinopathy [29]; however, Gomes et al. later identified abnormalities in the retinal microvasculature [22]. FFA has provided new insights into retinochoroidal impairment. Waszczykowska et al. identified vascular abnormalities in 55% of SSc cases using the FFA method [30]. The introduction of optical coherence tomography (OCT) has enabled the detection of novel findings in systemic diseases. Studies on SSc patients have reported significant choroidal thinning compared to controls [6,31]. A major advancement in detecting subtle microvascular changes in these patients was achieved through OCT angiography. In 2019, Ranjbar et al. were the first to demonstrate decreased perfusion across all choroidal layers in the submacular region in patients with SSc using optical coherence tomography angiography (OCTA) [32]. Mihailovic et al. reported a decrease in choriocapillaris vessel density, which was evident even in the early stages of SSc [33]. Additionally, they found a negative correlation between skin score and superficial capillary plexus vessel density, reinforcing the notion that microvascular changes play a significant pathogenic role in SSc [32]. In a previous study, we assessed the retinas of SSc patients using OCTA and identified abnormalities in the retinal and choroidal microvasculature [9]. In our current study, no significant difference was observed in the elastography values of RCS between SSc patients and the control group. This may be due to the choroid’s extensive vasculature and the tendency of SSc-related microvascular changes to primarily affect small capillaries and arterioles, potentially preserving retinal and choroidal elasticity in the early stages. Longer follow-up studies are needed to confirm this. However, consistent with previous studies, we observed a strong association between the presence of DUs and increased stiffness values of RCS.

ON involvement in SSc is typically regarded as a secondary manifestation linked to glaucoma, as several studies have investigated the relationship between SSc and the prevalence of glaucoma [34]. Gomes et al. observed open-angle glaucoma in 11.1% of their study’s participants, a prevalence higher than that in the general population [22]. Elastographic measurements of OD and ON in individuals with glaucoma exhibit considerable variability, likely influenced by factors by factors such as the type, duration, and severity of SSc. For example, studies conducted by Dikici et al. and Unal et al. reported that ON elasticity values were significantly higher in glaucoma patients compared with the controls [15,35]. In contrast, research by Agladioglu et al. and Hekimoglu et al. on glaucoma patients found no significant differences in the stiffness of the ON and OD between the glaucoma and control groups [36,37]. In this study, we found no statistical difference between SSc patients and the control group in the elasticity of the optic nerve and optic disc.

Orbital fat is gaining recognition for its significant role in the development of various inflammatory diseases, mainly due to its immunological properties and strategic anatomical location [38], such as in Graves’ disease, orbital cellulitis, glaucoma, and pseudotumor [39,40,41]. Currently, no direct studies in the literature have examined changes in RBFT in patients with SSc. Our study revealed increased stiffness in RBFT among SSc patients. Moreover, the elasticity measurement of RBFT demonstrates a high diagnostic value in identifying SSc, with a sensitivity of 79% and specificity of 77%.

Retinoids in orbital fat can directly influence orbital fibroblasts, promoting the expression of genes associated with inflammation [42]. For example, TSH receptor antibodies (TRAb) are a hallmark of Graves’ disease, and their production is thought to be both an initial trigger and a crucial factor in the development of Graves’ orbitopathy. When TRAb binds to the TSH receptor on orbital fibroblasts, it initiates an immune response, leading to the infiltration of activated B and T cells, as well as CD34+ fibrocytes from the bone marrow. These fibrocytes subsequently differentiate into myofibroblasts or adipocytes [43].

SSc is a multi-system autoimmune disease affecting various organs and systems. Accordingly, we compared our elastography values with clinical parameters that are more commonly observed and have greater prognostic significance. Pulmonary function parameters (FVC and DLCO) were included in the analysis because pulmonary fibrosis and ILD are major causes of morbidity and mortality in SSc, and the early detection of pulmonary involvement is crucial [44]. We found no significant relationship between the elastography or velocity measurements and FVC or DLCO and disease duration (both post-first RP and post-non-Raynaud’s symptoms) in patients with SSc. PHT is a leading cause of death in SSc, associated with vascular remodeling and increased stiffness of the pulmonary vasculature [45]. Given the ability of SWE to detect changes in the mechanical properties of tissue, we hypothesized that microvascular dysfunction and fibrosis associated with PHT might influence the SWE values. However, no significant statistical relationship was found between the presence of PHT and the elastography findings. Gastrointestinal involvement, particularly esophageal reflux, is highly prevalent in SSc patients, affecting up to 90% of cases, and reflects gastrointestinal fibrosis and dysmotility [46]. In this study, we found a significant relationship between OD elasticity and gastroesophageal reflux. Finally, DU represent a hallmark of severe microangiopathy and vascular injury in patients with SSc [47]. A significant association was found between the RCS velocity and gastroesophageal reflux.

This study has several limitations. Firstly, the sample size was relatively small, which may affect the statistical power of the findings. Secondly, the study cohort consisted predominantly of female participants, which may introduce a gender-related bias. Thirdly, the research was conducted at a single center and was not designed as a community-based study, thus limiting the generalizability of the results to the broader population. Fourthly, since there were no patients with cardiac or renal complications and active DUs in our study, the correlation of these parameters with SWE findings could not be evaluated. Lastly, intra-observer variability was not assessed, as all SWE measurements were performed by a single radiologist.

## 5. Conclusions

In this study, we observed an increased stiffness in the RBFT of SSc patients. To our knowledge, this is the first evidence suggesting that SSc can affect the RBFT. A strong association was found between OD elasticity and the presence of gastroesophageal reflux, as well as between DUs and increased stiffness of the RCS complex. The early identification of microcirculatory changes in the eye may facilitate earlier diagnosis and more effective management of the disease. In conclusion, these results suggest that orbital evaluation through elastography holds significant potential as a diagnostic tool for SSc and its associated complications in the future. Further research is needed to validate this finding and explore its relationship with the disease.

## Figures and Tables

**Table 1:** Demographic information of participants.

Parameters	Patients		Control		t/x^2^	*p* *
	$\bar{X}$ ± S	Lower-Upper	$\bar{X}$ ± SD	Lower-Upper		
Age, years	51.21 ± 13.06	21–71	50.37 ± 14.37	20–70	0.44	0.515
BMI, kg/m^2^	25.86 ± 3.58	19.81–34.21	25.54 ± 3.18	21.22–32.05	0.36	0.717
FVC	90.34 ± 12.52	62–100	-	-		-
DLCO	91.00 ± 13.52	60–102	-	-	-	-
Post-first RP disease duration, month	41.31 ± 27.99	12–140	-	-	-	-
Post-non-Raynaud’s symptoms disease duration, month	28.34 ± 26.71	4–128	-	-	-	-
Gender	Female = 27	Male = 2	Female = 26	Male = 4	0.67	0.413
Disease Type	Diffuse = 22	Limited = 7	-	-	-	-
PAD	Yes = 1	None = 28	-	-	-	-
ILD	Yes = 5	None = 24	-	-	-	-
DU	Yes = 16	None = 9	-	-	-	-
Antibody Type	anti-Scl-70 = 8	Anti centromere = 21	-	-	-	-
Reflux	Yes = 15	None = 14	-	-	-	-
PHT	Yes = 6	None = 23	-	-	-	-

Descriptive characteristics were given as mean ± standard deviation (SD) (minimum–maximum). Abbreviations: FVC: forced vital capacity, DLCO: diffusing capacity of the lungs for carbon monoxide, PAD: peripheral arterial disease, ILD: interstitial lung disease, DU: digital ulcer; PHT: pulmonary hypertension RP: Raynaud’s phenomenon. * *p* < 0.05 indicates statistical significance.

**Table 2:** Coefficient of variance for repeated elastography measurements.

Measurement Variables	Mean Value (Mean)	Mean Standard Deviation (SD)	Mean Coefficient of Variation (CV, %)
Elasticity RCS	94.34	1.31	1.42
Elasticity OD	60.57	0.22	0.49
Elasticity ON	19.22	0.16	1.02
Elasticity RBF	7.39	0.10	1.50
Velocity RCS	5.72	0.05	0.84
Velocity OD	4.32	0.03	0.68
Velocity ON	2.56	0.02	0.68
Velocity RBF	1.51	0.01	0.68

SD: standard deviation. CV: coefficient of variation.

**Table 3:** Comparison of elastography values of patient and control groups.

Elastography Parameters	Subgroup	N	$\bar{X}$ ± SD	t	*p* *	D
Elasticity RCS	Patient	29	91.62 ± 24.34	1.36	0.179	-
	Control	30	102.50 ± 35.83			
Elasticity OD	Patient	29	60.37 ± 35.59	0.06	0.956	-
	Control	30	60.88 ± 35.18			
Elasticity ON	Patient	29	19.67 ± 9.27	0.39	0.698	-
	Control	30	18.86 ± 6.39			
Elasticity RBF	Patient	29	8.78 ± 2.75	4.74	<0.001 *	1.23
	Control	30	6.08 ± 1.48			
Velocity RCS	Patient	29	5.61 ± 0.95	0.62	0.538	-
	Control	30	5.78 ± 1.15			
Velocity OD	Patient	29	4.31 ± 1.44	0.04	0.967	-
	Control	30	4.32 ± 1.40			
Velocity ON	Patient	29	2.66 ± 0.63	1.44	0.156	-
	Control	30	2.46 ± 0.41			
Velocity RBF	Patient	29	1.60 ± 0.37	2.13	0.037 *	0.53
	Control	30	1.44 ± 0.21			

Abbreviations: RCS: retina–choroid–sclera, ON: optic nerve, OD: optic disc, RBF: retrobulbar fat tissue, SD: standard deviation. * *p* < 0.05 indicates statistical significance.

**Table 4:** Comparison of elastography values of patient groups according to disease type.

Elastography Parameters	Subgroup	N	$\bar{X}$ ± SD	t	*p* *
Elasticity RCS	Diffuse	22	91.10 ± 24.07	0.20	0.841
	Limited	7	93.27 ± 27.09		
Elasticity OD	Diffuse	22	61.09 ± 35.09	0.19	0.852
	Limited	7	58.13 ± 39.89		
Elasticity ON	Diffuse	22	19.36 ± 9.97	0.31	0.762
	Limited	7	20.61 ± 7.15		
Elasticity RBF	Diffuse	22	8.56 ± 1.91	0.76	0.456
	Limited	7	9.47 ± 4.66		
Velocity RCS	Diffuse	22	5.57 ± 0.96	0.37	0.718
	Limited	7	5.73 ± 0.97		
Velocity OD	Diffuse	22	4.45 ± 1.54	0.97	0.340
	Limited	7	3.84 ± 1.07		
Velocity ON	Diffuse	22	2.69 ± 0.69	0.45	0.657
	Limited	7	2.56 ± 0.44		
Velocity RBF	Diffuse	22	1.60 ± 0.40	0.04	0.972
	Limited	7	1.61 ± 0.28		

Abbreviations: RCS: retina–choroid–sclera, ON: optic nerve, OD: optic disc, RBF: retrobulbar fat tissue, SD: standard deviation. * *p* < 0.05 indicates statistical significance.

**Table 5:** Disease durations and FVC, DLCO, and elastography values of the patient group between correlation.

Scale	FVC	DLCO	Post-First RP Disease Duration	Post-Non-Raynaud’s Symptoms Disease Duration
Elasticity RCS	−0.30	−0.22	0.22	0.15
Elasticity OD	−0.22	−0.18	−0.01	−0.06
Elasticity ON	−0.12	−0.09	−0.06	−0.04
Elasticity RBF	0.02	0.05	−0.10	−0.15
Velocity RCS	−0.33	−0.28	−0.05	−0.11
Velocity OD	−0.12	−0.06	−0.13	−0.11
Velocity ON	0.03	0.04	−0.20	−0.15
Velocity RBF	−0.06	0.01	−0.28	−0.32

Abbreviations: RCS: retina–choroid–sclera, ON: optic nerve, OD: optic disc, RBF: retrobulbar fat tissue, RP: Raynaud’s phenomenon. Values are presented as Pearson’s correlation coefficients (r).

**Table 6:** Comparison of elastography values according to the presence of reflux in the patient group.

Elastography Parameters	Subgroup	N	$\bar{X}$ ± SD	T	*p* *	d
Elasticity RCS	Yes	15	95.77 ± 27.19	0.95	0.352	-
	None	14	87.18 ± 20.95			
Elasticity OD	Yes	15	76.00 ± 36.88	2.71	0.012 *	1.01
	None	14	43.63 ± 26.06			
Elasticity ON	Yes	15	21.43 ± 7.66	1.06	0.298	-
	None	14	17.78 ± 10.69			
Elasticity RBF	Yes	15	8.72 ± 3.29	0.13	0.901	-
	None	14	8.85 ± 2.15			
Velocity RCS	Yes	15	5.80 ± 0.99	1.13	0.267	-
	None	14	5.40 ± 0.89			
Velocity OD	Yes	15	4.88 ± 1.43	2.42	0.023 *	0.90
	None	14	3.69 ± 1.22			
Velocity ON	Yes	15	2.60 ± 0.50	0.49	0.627	-
	None	14	2.72 ± 0.77			
Velocity RBF	Yes	15	1.54 ± 0.27	0.90	0.375	-
	None	14	1.67 ± 0.45			

Abbreviations: SD: standard deviation, RCS: retina–choroid–sclera, ON: optic nerve, OD: optic disc, RBF: retrobulbar fat tissue. * *p* < 0.05 indicates statistical significance.

**Table 7:** Comparison of elastography values according to the presence of digital ulcer in the patient group.

Elastography Parameters	Subgroup	N	$\bar{X}$ ± SD	T	*p* *	D
Elasticity RCS	Yes	16	104.41 ± 21.01	3.83	0.001 *	0.87
	None	13	75.88 ± 18.56			
Elasticity OD	Yes	16	71.04 ± 39.39	1.87	0.072	
	None	13	47.24 ± 26.02			
Elasticity ON	Yes	16	22.23 ± 10.16	1.71	0.099	-
	None	13	16.51 ± 7.19			
Elasticity RBF	Yes	16	8.98 ± 3.11	0.43	0.674	-
	None	13	8.54 ± 2.32			
Velocity RCS	Yes	16	6.11 ± 0.77	3.87	0.001 *	0.88
	None	13	4.99 ± 0.78			
Velocity OD	Yes	16	4.68 ± 1.50	1.53	0.137	-
	None	13	3.86 ± 1.29			
Velocity ON	Yes	16	2.83 ± 0.67	1.70	0.101	-
	None	13	2.44 ± 0.53			
Velocity RBF	Yes	16	1.64 ± 0.32	0.55	0.56	-
	None	13	1.56 ± 0.42			

Abbreviations: SD: standard deviation, RCS: retina–choroid–sclera, ON: optic nerve, OD: optic disc, RBF: retrobulbar fat tissue. * *p* < 0.05 indicates statistical significance.

**Table 8:** Assessment of the diagnostic performance of elastography findings in the diagnosis of systemic sclerosis.

Risk Factor	AUC (%95)	Cut Off	*p* *	Sensitivity (%)	Specificity (%)
Elasticity RCS	0.427 (0.279–0.577)	94.25	0.336	44.8	43.3
Elasticity OD	0.483 (0.332–0.633)	56.10	0.820	51.0	50.0
Elasticity ON	0.508 (0.358–0.658)	18.75	0.915	52.0	53.0
Elasticity RBF	0.870 (0.782–0.952)	7.35	<0.001 *	79.0	77.0
Velocity RCS	0.462 (0.313–0.611)	5.67	0.617	48.0	50.0
Velocity OD	0.482 (0.332–0.632)	4.37	0.814	52.0	50.0
Velocity ON	0.582 (0.434–0.730)	2.50	0.278	59.0	57.0
Velocity RBF	0.623 (0.479–0.767)	1.51	0.105	59.0	60.0

Abbreviations: AUC: area under the curve. * *p* < 0.05 indicates statistical significance.

## Data Availability

The raw data supporting the conclusions of this article will be made available by the authors upon reasonable request.
